# Limiting factors in single particle cryo electron tomography

**DOI:** 10.5936/csbj.201207002

**Published:** 2012-07-01

**Authors:** Mikhail Kudryashev, Daniel Castaño-Díez, Henning Stahlberg

**Affiliations:** a Center for Cellular Imaging and NanoAnalytics (C-CINA), Biozentrum, University of Basel, Mattenstrasse 26, CH-4058 Basel, Switzerland

## Abstract

Modern methods of cryo electron microscopy and tomography allow visualization of protein nanomachines in their native state at the nanometer scale. Image processing methods including sub-volume averaging applied to repeating macromolecular elements within tomograms allow exploring their structures within the native context of the cell, avoiding the need for protein isolation and purification. Today, many different data acquisition protocols and software solutions are available to researchers to determine average structures of macromolecular complexes and potentially to classify structural intermediates. Here, we list the density maps reported in the literature, and analyze each structure for the chosen instrumental settings, sample conditions, main processing steps, and obtained resolution. We present conclusions that identify factors currently limiting the resolution gained by this approach.

## Introduction

The combination of vitreous preservation of biological samples [1] with 3D electron tomographic imaging [2] has opened a unique opportunity to study living matter at nanometer scale [3]. Importantly, in addition to observing the ultrastructure of cells, it is also possible to perform structural analysis of large macromolecules and their complexes in situ. Since the inherent contrast of cryo-preserved material is low, a large number of electron microscopy datasets of identical specimens is needed to allow statistical image processing with specialized algorithms and methods. This approach is fundamentally similar to single particle cryo-electron microscopy (cryo-EM) [4], except that each tomographic sub-volume is a 3-dimensional dataset with anisotropic resolution, and that the particles are imaged in a crowded cellular context rather than isolated in solution. The process of producing an average structure is commonly referred to as “cryo electron tomography sub-volume averaging”, or CET SVA. The typical resolution that may be achieved by CET SVA in situ is 2-6 nm, dependent upon on a number of factors. We here discuss four major sources of resolution degradation: 1) sample thickness, 2) cryo-EM instrumentation, 3) sample heterogeneity, and the 4) effect of the contrast transfer function of the instrument.

First, the quality of cryo-EM images worsens with increasing electron density thickness of the sample. The fraction of inelastically scattered electrons increases with growing sample thickness, degrading the resolution of structures derived from SVA. For the commonly used intermediate voltage transmission electron microscopes, operating at electron acceleration voltages of 200 or 300 kV, the sample thickness limit for tomographic imaging of biological specimens is around 0.5 – 1 µm. Second, the choice of cryo-EM instrumentation is important: electrons accelerated with higher voltages are capable of penetrating thicker samples; electron energy loss imaging filters eliminate inelastically scattered electrons of lower energy, thereby improving the signal to noise ratio of the resulting images. The point resolution of modern electron microscopes is in the angstrom range, while the resolution of CET SVA is so far typically in nanometer range so this aspect currently does not limit the resolution. Thirdly, large protein complexes in the native context are subject to flexibility originating from external forces and from their own structural heterogeneity. Sub-volume averaging requires all volumes to contain structures in exactly the same conformation. Structural variations among sub-volumes would otherwise blur the calculated average, thereby reducing the resolution. Fourth, the low contrast in cryo-EM images is usually compensated for by the instrument's operator by defocusing the microscope's objective lens, which introduces strong oscillations to the instrument's contrast transfer function (CTF) that have to be accounted for during image processing (see below).

Typical objects approached by CET SVA are large protein complexes inside intact bacterial [5–8] or eukaryotic cells [9–11]; large protein or protein-DNA complexes in cryo-sectioned cells or tissues [12–14]; viral cores [15–18] and outer surface proteins [19–21]; bacteriophages [22, 23]; microtubule bound proteins in situ [24–26] or in vitro [27]; membrane protein complexes in simplified systems such as lipid vesicles [28] or isolated membrane fractions [29] and proteins and their complexes that have a preferential orientation on the EM support [30, 31].

The popularity of CET SVA is growing fast, and the number of laboratories in the world with access to the expensive instrumentation and image processing expertise is rising rapidly. Although progress in single particle electron tomography has recently been reviewed [32], here we use the rapidly increasing number of publications to present a comprehensive analysis of recently published structures and employed imaging parameters from cryo electron tomography and sub-volume averaging.

## Overview

Table S1 summarizes the acquisition parameters and resulting resolutions of 123 structures solved by CET SVA that were found by literature searches (Using http://pubmed.gov and searching for “cryo electron tomography” and “electron cryotomography” before 15 Feb 2012) and contained structural analysis (99 entries, some examining more than one structure). We incorporated the image acquisition parameters, such as microscope accelerating voltage, applied objective lens defocus, tomographic angular coverage, presence of energy filtration, etc with the properties of the sample, such as estimated ice thickness and the properties of the protein complex of interest, such as number of averaged particles, symmetry and estimated maximal linear dimension.

**Figure 1:**
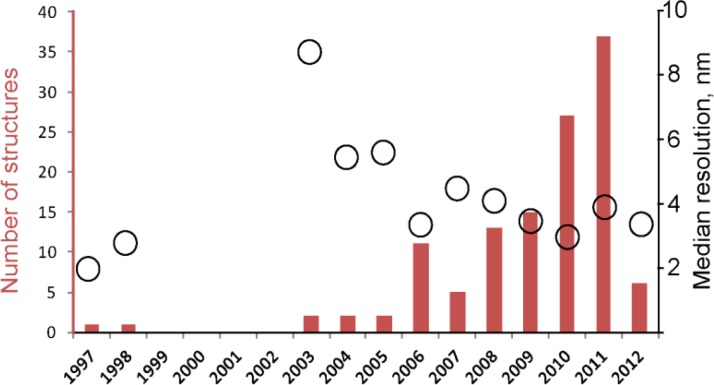
Number of structures solved by CET and sub-tomogram averaging (red bar graph) and average improvement in resolution (black circles).

The number of structures solved by sub volume averaging grows year from year: while only 8 structures had been analyzed before 2006, 44 structures were reported between 2006 and 2009; and 37 structures were reported in 2011 alone (Figure 1). As seen from Figure 1, not only does the number of structures grow, but also the resolution of these structures tends to improve.

We further performed a simple statistical analysis of acquisition parameters by correlation of values from columns of Table S1. Table 1 illustrates relationships between publication year and resolution and other experimental parameters. The resolution of the reconstruction was in most of cases assumed as that where the signal to noise ratio in frequency space was above a defined threshold. Resolution is usually defined as that were the Fourier Shell Correlation curve between averages produced by two sub-halves of the dataset intersects with the horizontal “0.5” threshold, or with a threshold of “0.143” [33], or yet other information criteria [34].

**Table 1:** Correlation coefficients (cc) between various data acquisition parameters and the publication year, as well as the resolution value. A positive cc value, as for example for the electron dose of 0.34 with the year, indicates that in later years higher electron doses were applied, while a correlation value of -0.28 between the number of particles and the achieved resolution indicates that with more particles generally a better (smaller) resolution value was reached. Relationships with high positive or negative correlation coefficients are highlighted with colors.

	Correlation with publication year	Correlation with Resolution Value
Number of particles	0.01	-0.28
Symmetry order	-0.15	0.21
Number of asymmetric units	0.02	-0.27
Number of classes	0.10	-0.14
Publication year	1.00	-0.13
Pixel size	-0.06	0.07
Camera size	0.25	0.05
Acceleration voltage	0.03	-0.27
Minimal underfocus	0.03	0.60
Presence of an energy imaging filter	0.25	0.12
Angular coverage	0.19	-0.22
Electron dose	0.34	0.30
Ice thickness	0.14	0.33
Largest linear sample size	0.12	0.44

There is a positive correlation detected between the publication year and camera size and presence of the imaging filter, which is an indication of improvement in instrumentation used by structural biological laboratories. Interestingly, there is no correlation between year and number of acquired particles, which suggests that there is no significant increase in automation of data collection noticeable. As mentioned before, the resolution of structures analyzed by CET improved with time.

## Factors affecting resolution

Higher resolution of the resulting structure was achieved when processing more particles. Table 1 here shows a correlation value between the number of particles and the resolution value of cc=-0.28, indicating that with more particles the number reported resolution for the resolution value became smaller (i.e., better resolution was reported). This trivial relationship illustrates the effect of improving the signal to noise ratio of the final reconstruction by increasing both the number particles and asymmetric units, when symmetric particles were processed. Interestingly, particles with higher symmetry on average yielded worse resolution (cc=0.21). This phenomenon may be related to a positive correlation (cc=0.14) with symmetry order and the linear size of the samples, suggesting that particles with higher symmetry are usually larger and may be more flexible than non-symmetric particles. Indeed, there is a high positive correlation between resolution value and linear size of the protein complex (cc=0.44), suggesting that despite being less visible in the raw cryo-EM images, smaller particles usually allowed for imaging parameter settings that led to higher resolution. Moreover, a larger quasi-symmetric protein assembly often deviates from ideal symmetry, when the particle is suffering from sample preparation influences, like flattening in a thinner ice layer. For example, datasets from bacterial flagellar motors of spirochetes processed using C16 symmetry [5, 35] have worse resolution than structures obtained without any symmetry assumptions [6], although a substantially increased number of particles were included in the asymmetric reconstruction. The degree of flexibility of a multi protein complex has to be analyzed carefully, as even fairly large assemblies may be studied at relatively high resolution by CET and SVA [36–38]. If a sufficiently large dataset is available, sample heterogeneity can be evaluated computationally by using classification of the aligned particles, wherein different particles or different conformations of the particles should be re-assigned to separate classes. This then reduces the in-class variation, thereby allowing higher resolution of the reconstruction in case of sufficient number of particles remaining in the class. Table 1 also shows a correlation (cc=-0.14) between the resolution and number of used classes. Note that this suggest a tradeoff between the potential resolution increase contributed by averages of more heterogeneous data sets (classes) and the resolution loss derived from the reduced number of particles in each averaged set. Thus, similarly to the relaxation of symmetry assumption, the use of classification techniques requires the collection of larger data sets.

Among the factors limiting resolution, ice thickness plays an important role: thicker ice layers contribute larger fractions of inelastically scattered electrons, thus decreasing the image quality. Researchers interested in protein complexes embedded in thicker ice layers on average apply higher electron dose (cc = 0.37), use higher accelerating voltage (cc = 0.33), image filtration (cc = 0.14) and higher objective lens underfocus (cc = 0.43), which further limits the resolution (see below).

An increase in the applied electron dose for collecting one dataset improves the signal to noise ratio, but also results in dose-dependent beam-induced sample destruction. This leads to a loss in resolution, and may also induce sample drift [39]. Certain resolution targets tolerate sample destruction to different extents. Total electron doses of 70 e/A^2^ allowed a resolution of 2 nm in a number of cases, while applying 200 e/A^2^ allowed at best a resolution in the range of 5 nm (Table S1).

Angular coverage determines the point spread function of the resulting tomograms and thus the sub-volumes to be aligned in CET SVA. For single tilt tomography, angular coverage defines the “missing wedge” of information [2] in the Fourier Space, while for dual tilt tomography the information is missing inside a cone. It is interesting to consider the effect of electron dose-related damage to the sample in Fourier space. The higher the angular coverage, the greater the Fourier volume is sampled for a given electron dose, so that over-exposure of low-resolution Fourier voxels along the tilt axis is minimized. While dual tilt-axis tomography is better suited to spread the beam damage over a larger Fourier volume than single tilt-axis tomography, both data collection schemes still massively over-expose the Fourier voxels along the one or two tilt axes in Fourier space with repeated electron beam exposure. A conical tilt geometry tomography data collection scheme [40] would minimize this over-exposure of Fourier voxels, and would optimally spread the allowed electron dose in Fourier space. The price for this optimization, however, would be increased effective electron density thickness as every image would be acquired for a tilted specimen, so that one non-tilted and a few low-angle tilted projection images should also be collected. Today's sample stage implementations on some existing electron microscopes (e.g. FEI Titan Krios) suggest that implementation of conical tilt tomography may be performed. Table 1 shows a negative correlation between resolution and angular coverage, meaning that higher resolution is on average achieved by higher angular coverage. In addition to the arguments given above, this may also be a consequence resulting from difficulties in the alignment of particles having a missing wedge. Even with specific algorithms designed to tackle this situation [41], it is not clear if complete compensation of the information lost in the missing wedge can be achieved for individual tomography sub-volumes, where oversampling of low-resolution data is limited by the allowed electron dose. Another possible hypothesis is that researchers try to use higher angular coverage for thinner ice layers that allow higher contrast also on highly tilted specimens. However, our analysis showed no correlation between angular coverage and ice layer thickness.

**Figure 2:**
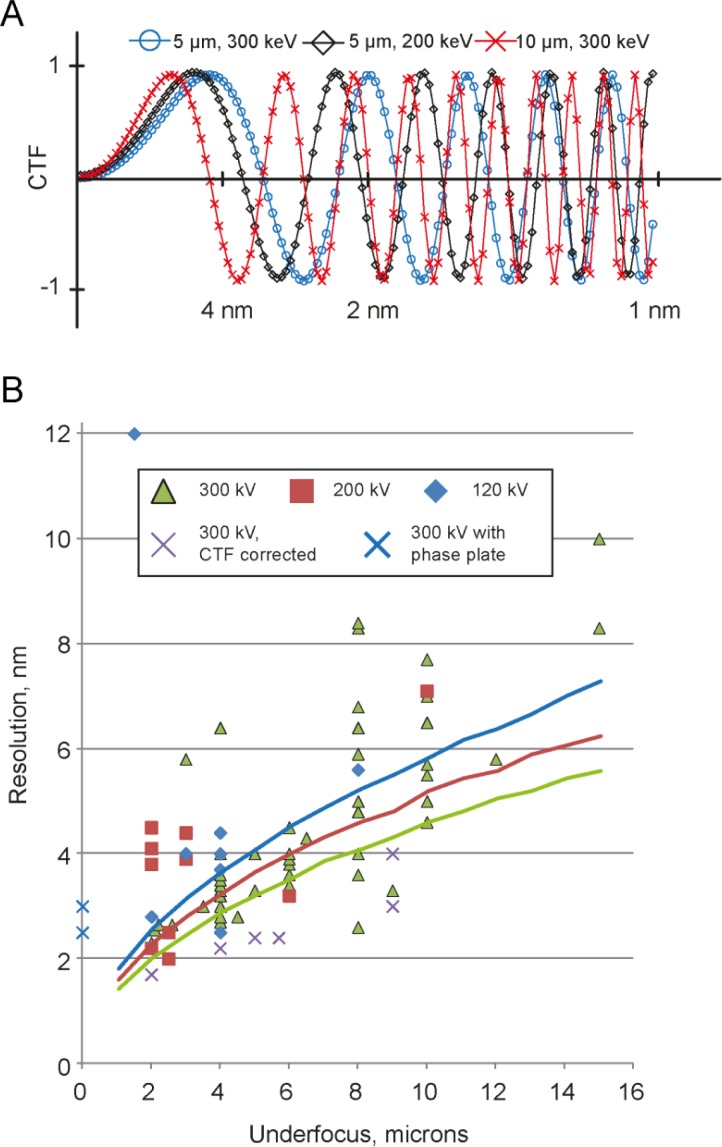
(A) Examples of CTF profiles for different conditions of underfocus and accelerating voltage. (B) Achieved resolution and applied defocus values for various projects listed in Table S1, sorted by electron acceleration voltages of 120, 200, and 300kV. Curves indicate the “first zero” of CTF depending on underfocus and accelerating voltage: 120 kV – blue, 200 kV – red, 300 kV – green.

Our analysis showed the strongest correlation between the resolution and the applied objective lens underfocus. This applied underfocus in combination with the electron acceleration voltage is the main determinant of the profile of the phase contrast transfer function, which is a sinusoidal function modulating the frequency components of the projected EM image [42] (Figure 2A). In addition to the profiles plotted in Figure 2A, the CTF is dampened at higher frequencies by an envelope function that originates from the limited spatial coherence and energy monochromaticity of the electron beam, and from several other factors, like specimen movement, etc. Both, the CTF's oscillation frequency and the strength of the CTF's envelope decay are dependent on the applied underfocus. Higher defocusing (underfocussing) of the EM's objective lens provides more low-frequency contrast at the expense of reduced contrast transfer at higher frequencies. Use of higher accelerating voltage extends the CTF into the high resolution area, improves sample penetration depth due to a lower inelastic scattering cross section of faster electrons, but also reduces the sample contrast due to a reduced elastic scattering cross section. In addition, most commonly used image recording media such as CCD cameras will be affected by a worsening point spread function in the recording device when exposed to higher electron beam energies [43]. Single particle cryo-EM typically determines the structure of protein complexes from cryo-EM 2D projection images, where the CTF can relatively easily be corrected and the protein structures recovered. For cryo-electron tomography, however, CTF correction is not yet a routine process (see below). The resolution of 3D reconstructions from CET SVA is therefore in most cases limited to the first zero crossing of the CTF. Figure 2B shows the achieved resolution as a function of applied underfocus for structures listed in Table S1, acquired at accelerating voltages of 120, 200 and 300 kV. While a few published structures report to have overcome the “first zero” limit of the CTF, without proper CTF correction the reliability of the frequencies in the resolution range beyond the first Thon ring is questionable.

## Correction of the Contrast Transfer Function

A direct translation of CTF correction methods from single particle methodology [4] to electron tomography is not feasible for two reasons:

First, for thick cryo samples there is a defocus gradient along the axis of the electron beam. The image formation model provides a good framework for the derivation of a correcting step for projection images of thin samples, as in this assumption, the obtained image truly corresponds to the projection image of the object convoluted with a point spread function that remains constant in the image definition domain. Thus, suitable deconvolution algorithms can recover the initial image, possibly incorporating some processing, such as Wiener filtering, to address the low SNR of cryo-EM data and the vanishing information around the zeros of the CTF. This approach can be extended for the correction of CTF on images of tilted samples, as is customarily done in electron-crystallography [44–46]. However, for thick samples, where extreme vertical points of the imaged specimen are placed in different defocus distances, the treatment of the collected signal as a convolution of true signal with a simply describable transfer function is no longer valid. Correcting such images with a simple CTF correction without taking the defocus variations throughout the thicker specimen into account is likely to result in artifacts and thereby limit the achievable resolution. Tilting of the samples as done in tomography increases the gradient proportional to the amount of tilting.

Secondly, while it is possible to determine the defocus of the micrograph for a single particle data acquisition by fitting Thon rings [47], in tomography the applied electron dose has to be divided among multiple images (typically 40-120), which reduces the signal to noise ratio in the individual images such that detection of a defocus value from a single micrograph becomes a challenging task. For tilted images a more complicated tilted CTF has to be taken into account [44]. Tomography data acquisition routines include potential focusing steps before the recording of every image. However, the focusing is not always precise [37]. To date, several implementations of CTF corrections are available [48–50], and have been shown to be capable of resolution improvement for samples thin ice layers (Figure 2B, magenta crosses).

A recently proposed method to partially circumvent the CTF barrier is “focal pair tomography” [51]. This includes acquisition of two consecutive tilt series from the same object, first at low underfocus in order to get high resolution information followed by a tilt series at high underfocus in order to collect data at high contrast. Two approaches were proposed: acquiring focal pairs from every view simultaneously, or the consecutive acquisition of two full tilt series. In the second case the sample receives a considerable electron dose before the start of the second tilt series acquisition, which causes the destruction of high resolution details (that may be recovered from firstly acquired tilt series) and results in sample movement, which may be compensated for with image processing. A data processing approach that uses the information from both tilt series allows achieving slightly higher resolution than when applying intermediate underfocusing conditions on synthetic [51] and on real data [Kudryashev et at, unpublished data].

Another powerful method to increase resolution and contrast in electron microscopy is by employing a so-called phase plate during data collection. Phase plates allow close-to-focus imaging of cryo preserved biological material with significant contrast. Nevertheless, the defocus gradient through thicker samples or across tilted specimens leads to sample areas that are imaged with an oscillating CTF even when utilizing a phase plate. The principles and current challenges are reviewed elsewhere [52]. For tomographic data collection and sub-volume averaging phase plates provide a stunning advantage: since every particle has both high contrast and fine details, the requirements for the number of collected particles are significantly reduced [38]. Using this technology, the structure of the Epsilon15 Bacteriophage was determined at 25 Å resolution with application of icosahedral symmetry from 50 particles, or at 30 Å resolution without application of symmetry from 95 particles (also see Figure 2B, blue crosses). Although to date the resolution of reconstructions from SVA with a phase plate is still slightly lower than of defocused datasets, fascinating details of single membrane proteins in whole mounted frozen cells in phase plate tomograms are very promising [53].

## Conclusion

Overall, CET SVA demonstrates a consistent trend both in the increase of publications and in quality of the determined structures. More exciting research is expected to come. The quality of the instrumentation is also rapidly improving, which should further improve reconstruction quality. Finally, a significant improvement in resolution is expected from a broad application of contrast transfer function correction for cryo-electron tomography data processing.

## Supplementary Material

Limiting factors in single particle cryo electron tomography

## References

[1] Dubochet J, Adrian M, Chang JJ, Homo JC, Lepault J, et al. (1988) Cryo-electron microscopy of vitrified specimens. Q Rev Biophys 21: 129–228 3043536 10.1017/s0033583500004297

[2] Baumeister W (2002) Electron tomography: towards visualizing the molecular organization of the cytoplasm. Curr Opin Struct Biol 12: 679–684 12464323 10.1016/s0959-440x(02)00378-0

[3] Leis A, Rockel B, Andrees L, Baumeister W (2008) Visualizing cells at the nanoscale. Trends Biochem Sci 34(2): 60–70 19101147 10.1016/j.tibs.2008.10.011

[4] Frank J (2009) Single-particle reconstruction of biological macromolecules in electron microscopy--30 years. Quarterly reviews of biophysics 42: 139–158 20025794 10.1017/S0033583509990059 PMC2844734

[5] Murphy GE, Leadbetter JR, Jensen GJ (2006) In situ structure of the complete Treponema primitia flagellar motor. Nature 442: 1062–1064 16885937 10.1038/nature05015

[6] Liu J, Lin T, Botkin DJ, McCrum E, Winkler H, et al. (2009) Intact flagellar motor of Borrelia burgdorferi revealed by cryo-electron tomography: evidence for stator ring curvature and rotor/C-ring assembly flexion. J Bacteriol 191: 5026–5036 19429612 10.1128/JB.00340-09 PMC2725586

[7] Ortiz JO, Brandt F, Matias VR, Sennels L, Rappsilber J, et al. (2010) Structure of hibernating ribosomes studied by cryoelectron tomography in vitro and in situ. The Journal of cell biology 190: 613–621 20733057 10.1083/jcb.201005007 PMC2928015

[8] Khursigara CM, Lan G, Neumann S, Wu X, Ravindran S, et al. (2011) Lateral density of receptor arrays in the membrane plane influences sensitivity of the E. coli chemotaxis response. Embo J 30: 1719–1729 21441899 10.1038/emboj.2011.77 PMC3101988

[9] Brandt F, Carlson LA, Hartl FU, Baumeister W, Grunewald K (2010) The three-dimensional organization of polyribosomes in intact human cells. Molecular cell 39: 560–569 20797628 10.1016/j.molcel.2010.08.003

[10] Frenkiel-Krispin D, Maco B, Aebi U, Medalia O (2010) Structural analysis of a metazoan nuclear pore complex reveals a fused concentric ring architecture. Journal of molecular biology 395: 578–586 19913035 10.1016/j.jmb.2009.11.010

[11] Beck M, Forster F, Ecke M, Plitzko JM, Melchior F, et al. (2004) Nuclear pore complex structure and dynamics revealed by cryoelectron tomography. Science 306: 1387–1390 15514115 10.1126/science.1104808

[12] Daum B, Nicastro D, Austin J 2nd, McIntosh JR, Kuhlbrandt W (2010) Arrangement of photosystem II and ATP synthase in chloroplast membranes of spinach and pea. Plant Cell 22: 1299–1312 20388855 10.1105/tpc.109.071431 PMC2879734

[13] Al-Amoudi A, Castano-Diez D, Devos DP, Russell RB, Johnson GT, et al. (2011) The three-dimensional molecular structure of the desmosomal plaque. Proceedings of the National Academy of Sciences of the United States of America 108: 6480–6485 21464301 10.1073/pnas.1019469108 PMC3081036

[14] Scheffer MP, Eltsov M, Frangakis AS (2011) Evidence for short-range helical order in the 30-nm chromatin fibers of erythrocyte nuclei. Proc Natl Acad Sci U S A 108: 16992–16997 21969536 10.1073/pnas.1108268108 PMC3193215

[15] Plevka P, Battisti AJ, Junjhon J, Winkler DC, Holdaway HA, et al. (2011) Maturation of flaviviruses starts from one or more icosahedrally independent nucleation centres. Embo rep 12: 602–606 21566648 10.1038/embor.2011.75 PMC3128282

[16] Beniac DR, Melito PL, Devarennes SL, Hiebert SL, Rabb MJ, et al. (2012) The organisation of Ebola virus reveals a capacity for extensive, modular polyploidy. PLoS ONE 7: e29608 22247782 10.1371/journal.pone.0029608 PMC3256159

[17] Briggs JA, Riches JD, Glass B, Bartonova V, Zanetti G, et al. (2009) Structure and assembly of immature HIV. Proc Natl Acad Sci U S A 106: 11090–11095 19549863 10.1073/pnas.0903535106 PMC2700151

[18] Chang JT, Schmid MF, Rixon FJ, Chiu W (2007) Electron cryotomography reveals the portal in the herpesvirus capsid. Journal of virology 81: 2065–2068 17151101 10.1128/JVI.02053-06 PMC1797573

[19] White TA, Bartesaghi A, Borgnia MJ, de la Cruz MJ, Nandwani R, et al. (2011) Three-dimensional structures of soluble CD4-bound states of trimeric simian immunodeficiency virus envelope glycoproteins determined by using cryo-electron tomography. Journal of virology 85: 12114–12123 21937655 10.1128/JVI.05297-11 PMC3209358

[20] Liu J, Bartesaghi A, Borgnia MJ, Sapiro G, Subramaniam S (2008) Molecular architecture of native HIV-1 gp120 trimers. Nature 455: 109–113 18668044 10.1038/nature07159 PMC2610422

[21] Huiskonen JT, Hepojoki J, Laurinmaki P, Vaheri A, Lankinen H, et al. (2010) Electron cryotomography of Tula hantavirus suggests a unique assembly paradigm for enveloped viruses. Journal of virology 84: 4889–4897 20219926 10.1128/JVI.00057-10 PMC2863824

[22] Leo-Macias A, Katz G, Wei H, Alimova A, Katz A, et al. (2011) Toroidal surface complexes of bacteriophage varphi12 are responsible for host-cell attachment. Virology 414: 103–109 21489589 10.1016/j.virol.2011.03.020 PMC3095694

[23] Dai W, Hodes A, Hui WH, Gingery M, Miller JF, et al. (2010) Three-dimensional structure of tropism-switching Bordetella bacteriophage. Proc Natl Acad Sci U S A 107: 4347–4352 20160083 10.1073/pnas.0915008107 PMC2840154

[24] Bui KH, Sakakibara H, Movassagh T, Oiwa K, Ishikawa T (2009) Asymmetry of inner dynein arms and inter-doublet links in Chlamydomonas flagella. J Cell Biol 186: 437–446 19667131 10.1083/jcb.200903082 PMC2728406

[25] Nicastro D, Fu X, Heuser T, Tso A, Porter ME, et al. (2011) Cryo-electron tomography reveals conserved features of doublet microtubules in flagella. Proc Natl Acad Sci U S A 108: E845–853 21930914 10.1073/pnas.1106178108 PMC3198354

[26] Cyrklaff M, Kudryashev M, Leis A, Leonard K, Baumeister W, et al. (2007) Cryoelectron tomography reveals periodic material at the inner side of subpellicular microtubules in apicomplexan parasites. J Exp Med 204: 1281–1287 17562819 10.1084/jem.20062405 PMC2118598

[27] Cope J, Gilbert S, Rayment I, Mastronarde D, Hoenger A (2010) Cryo-electron tomography of microtubule-kinesin motor complexes. J Struct Biol 170: 257–265 20025975 10.1016/j.jsb.2009.12.004 PMC2856765

[28] Trepout S, Taveau JC, Benabdelhak H, Granier T, Ducruix A, et al. (2010) Structure of reconstituted bacterial membrane efflux pump by cryo-electron tomography. Biochimica et biophysica acta 1798: 1953–1960 20599691 10.1016/j.bbamem.2010.06.019

[29] Renken C, Hsieh CE, Marko M, Rath B, Leith A, et al. (2009) Structure of frozen-hydrated triad junctions: a case study in motif searching inside tomograms. J Struct Biol 165: 53–63 19028586 10.1016/j.jsb.2008.09.011 PMC2655133

[30] Dudkina NV, Kudryashev M, Stahlberg H, Boekema EJ (2011) Interaction of complexes I, III, and IV within the bovine respirasome by single particle cryoelectron tomography. Proc Natl Acad Sci U S A 108: 15196–15200 21876144 10.1073/pnas.1107819108 PMC3174662

[31] Liu J, Taylor DW, Krementsova EB, Trybus KM, Taylor KA (2006) Three-dimensional structure of the myosin V inhibited state by cryoelectron tomography. Nature 442: 208–211 16625208 10.1038/nature04719

[32] Schmid MF (2011) Single-particle electron cryotomography (cryoET). Adv Protein Chem Struct Biol 82: 37–65 21501818 10.1016/B978-0-12-386507-6.00002-6

[33] Rosenthal PB, Henderson R (2003) Optimal determination of particle orientation, absolute hand, and contrast loss in single-particle electron cryomicroscopy. Journal of molecular biology 333: 721–745 14568533 10.1016/j.jmb.2003.07.013

[34] van Heel M, Schatz M (2005) Fourier shell correlation threshold criteria. Journal of structural biology 151: 250–262 16125414 10.1016/j.jsb.2005.05.009

[35] Kudryashev M, Cyrklaff M, Wallich R, Baumeister W, Frischknecht F (2010) Distinct in situ structures of the Borrelia flagellar motor. J Struct Biol 169: 54–61 19699799 10.1016/j.jsb.2009.08.008

[36] Fu X, Walter MH, Paredes A, Morais MC, Liu J (2011) The mechanism of DNA ejection in the Bacillus anthracis spore-binding phage 8a revealed by cryo-electron tomography. Virology 421: 141–148 22018785 10.1016/j.virol.2011.08.028 PMC3939024

[37] Zanetti G, Riches JD, Fuller SD, Briggs JA (2009) Contrast transfer function correction applied to cryo-electron tomography and sub-tomogram averaging. Journal of structural biology 168: 305–312 19666126 10.1016/j.jsb.2009.08.002 PMC2806944

[38] Murata K, Liu X, Danev R, Jakana J, Schmid MF, et al. (2010) Zernike phase contrast cryo-electron microscopy and tomography for structure determination at nanometer and subnanometer resolutions. Structure 18: 903–912 20696391 10.1016/j.str.2010.06.006 PMC2925294

[39] Brilot AF CJ, Cheng A, Pan J, Harrison SC, Potter CS, Carragher B, Henderson R, Grigorieff N. (2012) Beam-induced motion of vitrified specimen on holey carbon film. J Struct Biol 177(3): 630–637 22366277 10.1016/j.jsb.2012.02.003 PMC3322646

[40] Radermacher M, Wagenknecht T, Verschoor A, Frank J (1987) Three-dimensional reconstruction from a single-exposure, random conical tilt series applied to the 50S ribosomal subunit of Escherichia coli. Journal of microscopy 146: 113–136 3302267 10.1111/j.1365-2818.1987.tb01333.x

[41] Gipson BR, Masiel DJ, Browning ND, Spence J, Mitsuoka K, et al. (2011) Automatic recovery of missing amplitudes and phases in tilt-limited electron crystallography of two-dimensional crystals, Physical review E, Statistical, nonlinear. and soft matter physics 84: 011916 10.1103/PhysRevE.84.011916 21867222

[42] Scherzer O (1948) Ein Elektronenoptischer Apochromat, Zeitschrift Fur Naturforschung Section a–a. J Phys Sci 3: 544–545

[43] Downing KH, Hendrickson FM (1999) Performance of a 2k CCD camera designed for electron crystallography at 400 kV. Ultramicroscopy 75: 215–233 9919710 10.1016/s0304-3991(98)00065-5

[44] Philippsen A, Engel HA, Engel A (2007) The contrast-imaging function for tilted specimens. Ultramicroscopy 107: 202–212 16989948 10.1016/j.ultramic.2006.07.010

[45] Henderson R, Baldwin JM, Downing KH, Lepault J, Zemlin F (1986) Structure of Purple Membrane from Halobacterium-Halobium - Recording, Measurement and Evaluation of Electron-Micrographs at 3.5 a Resolution. Ultramicroscopy 19: 147–178

[46] Mariani V, Schenk AD, Philippsen A, Engel A (2011) Simulation and correction of electron images of tilted planar weak-phase samples. Journal of structural biology 174: 259–268 21362479 10.1016/j.jsb.2011.02.008

[47] Thon F (1971) High Resolution Microscopy Using Special Apertures and Phase Plates. Journal of Electron Microscopy 20: 215

[48] Fernandez JJ, Li S, Crowther RA (2006) CTF determination and correction in electron cryotomography. Ultramicroscopy 106: 587–596 16616422 10.1016/j.ultramic.2006.02.004

[49] Winkler H (2007) 3D reconstruction and processing of volumetric data in cryo-electron tomography. J Struct Biol 157: 126–137 16973379 10.1016/j.jsb.2006.07.014

[50] Xiong Q, Morphew MK, Schwartz CL, Hoenger AH, Mastronarde DN (2009) CTF determination and correction for low dose tomographic tilt series. Journal of structural biology 168: 378–387 19732834 10.1016/j.jsb.2009.08.016 PMC2784817

[51] Kudryashev M, Stahlberg H, Castano-Diez D (2012) Assessing the benefits of focal pair cryo-electron tomography. Journal of structural biology 178: 88–97 22056466 10.1016/j.jsb.2011.10.004

[52] Nagayama K (2011) Another 60 years in electron microscopy: development of phase-plate electron microscopy and biological applications. Journal of Electron Microscopy 60 Suppl 1: S43–62 10.1093/jmicro/dfr037 21844600

[53] Fukuda Y, Fukazawa Y, Danev R, Shigemoto R, Nagayama K (2009) Tuning of the Zernike phase-plate for visualization of detailed ultrastructure in complex biological specimens. Journal of structural biology 168: 476–484 19732832 10.1016/j.jsb.2009.08.011

