# Supplementary material for: Limiting factors in single particle cryo electron tomography
Source: Comput Struct Biotechnol J. 2012 Jul 1;1:e201207002. doi: 10.5936/csbj.201207002 (PMC3962116; doi:10.5936/csbj.201207002)
Supplement: Limiting factors in single particle cryo electron tomography [file CSBJ-1-e201207002_SM0001.pdf]

Limiting factors in single particle cryo electron tomography.

Mikhail Kudryashev, Daniel Castaño-Díez and Henning Stahlberg

Supplemental Material

Protein complex	Origin	Reference	Publication Year	N part	Under–focus [µm]	Reso–lution [nm]	Pixel size [nm]	Classes	Sym–metry	Ice thick–ness [nm]	mag [x1000]	Camer a [pixels]	Acceler ating voltage [keV]	Energy filter used?	Angular coverage [deg]	Total electron dose $[e/A^2]$	Largest linear size [nm]	Dualt ilt used ?	Data processing software
Bacterial flagellar motor	*Borrelia burgdoferii*,flgJBb deletion mutant	[1]	2012	321	4 to 6	3.5	0.22	1	1	300	31	4096	300	no	128	100	100	no	[2]
Basal body triplet		[3]	2012	1644	9 to 24#	33	6.5	1	1	300	34	2048	300	yes	120	80	39	no	[4,5]
Ebola Virus capsid		[6]	2012		6 to 8			1	1		25 to 50		300		120	60		no	ARGOS
Marburg Virus		[7]	2011	68		2.6	0.49	3	1	100	27.5	2048	300	yes	120	100	77	no	[8]
Bacterial flagellar motor	*Borrelia burgdoferii*	[9]	2012	138	10	4.6	0.82	2	c16	300		2048	300	yes	120	200	100	no	[9]
Influenza virus		[10]	2012		4	2.5	0.78		c3			2048	120	yes	132	70	35	no	[11] and [12]
Inner dynein arms and inter-doublet links in	mouse respiratory cilia	[13]	2012	45	4 to 4.5			1	1		27	2048	200	yes	120		96	no	[12] and [5]
Flagellum	*Trypanosoma brucei*	[14]	2011		6 to 10			1	C1, longitudinal repeat	300	11 to 16	4096	200	yes	120	60-74	96	no	[15]
Actin-myosin contacts in muscle, freeze substituted		[16]	2010	515		3.5	0.69	7	C1, longitudinal repeat	50		2048	300	no	144	420	39	yes	[2]
	A. longum		2011	35	8 to 12	8.4	1.9	1	C16			2048	300	yes	120	200	100	part.	
	B. burgdorferi		2011	43	8 to 12	5	1.3	1	C16	300		2048	300	yes	120	200	100	part.	
	T. primitia		2006	20	10 to 18	7.7	1.9	1	C16	300		2048	300	yes	120	200	100	part.	
	H. hepaticus		2011	60	8 to 12	5	1.3	1	C13			2048	300	yes	120	200	100	part.	
	C. jejuni		2011	15	8 to 12	6.4	1.9	1				2048	300	yes	120	200	100	part.	
Bacterial flagellar motors	*H. gracilis*	[17]	2011	94	8 to 12	5.9	1.3	1				2048	300	yes	120	200	100	part.	[12] and [18]
	V. cholerae		2011	16	8 to 12	8.3	1.9	1				2048	300	yes	120	200	100	part.	
	S. enterica		2011	83	8 to 12	4.8	1.3	1		600		2048	300	yes	120	200	100	part.	
	E. coli		2011	46	8 to 12	5.9	1.3	1		600		2048	300	yes	120	200	100	part.	
	C. crescentus		2011	30	8 to 12	5.9	1.3	1		400		2048	300	yes	120	200	100	part.	
	H. neptunium		2011	27	8 to 12	6.8	1.5	1				2048	300	yes	120	200	100	part.	
Radial spokes	*Chlamydomonas flagella*	[19]	2011		6 to 8	3.3	1	1	C1, longitudinal repeat	350		2048	300	yes	130	100	96	no	
Procapsid possible packaging intermediates	Bacteriophage ϕ6	[20]	2011	120	4	4.4	0.78	4	C5	50	22		120	yes	132	70	50	no	[12]

Protein complex	Origin	Reference	Publication Year	N part	Under–focus [µm]	Reso–lution [nm]	Pixel size [nm]	Classes	Sym–metry	Ice thick–ness [nm]	mag [x1000]	Camer a [pixels]	Acceler ating voltage [keV]	Energy filter used?	Angular coverage [deg]	Total electron dose [e/A^2]	Largest linear size [nm]	Dualt ilt used ?	Data processing software
DNA ejection machinery	*Bacillus anthracis, phage 8a*	[21]	2011	2518	4 to 6	3.6	0.39	4	5 and 6	100		4096	300	no	128	100	200	no	[2]
Envelope Glycoproteins	Trimeric Simian Immunodeficiency Virus	[22]	2011	900-4800	2.5	2.5	0.41	10	C3	100	34	2048	200	yes	125	90	41	no	[23]
Doublet microtubules in flagella	Chlamydomonas and sea urchin sperm, drc Mutants	[24]	2011	1980	6 to 8	3.3	1	1	C1, longitudinal repeat	200		2048	300	yes	130	100	96	no	[25]
	Chlamydomonas and sea urchin sperm, pWT		2011	720	6 to 8	3.4	1	1	C1, longitudinal repeat	200		2048	300	yes	130	100	96	no	
	Chlamydomonas and sea urchin sperm, pWT		2011	210	6 to 8	3.9	1	1	C1, longitudinal repeat	200		2048	300	yes	130	100	96	no	
	Chlamydomonas and sea urchin sperm, in Flagella		2011	470	6 to 8	3.6	1	1	C1, longitudinal repeat	200		2048	300	yes	130	100	96	no	
	Chlamydomonas and sea urchin sperm, isolated DMT		2011	118	6 to 8	3.8	1	1	C1, longitudinal repeat	200		2048	300	yes	130	100	96	no	
Flagella	sea urchin sperm	[26]	2006		3.5 to 5	3	0.53	1	1	100		2048	300	yes	130	65	96	no	[12] and [5]
Flagella	sea urchin sperm	[27]	2005	56	3 to 6	5.8	0.59	1	1	200		2048	300	yes	130	100	96	no	[28]
	Bacteriophage P1 infecting e.coli	[29]	2011	219	9		0.78	3	5 and 6	300		2048	300	no	128	100	278	no	[2]
	Dengue virus	[30]	2011	30	3 to 5	4	0.78	1	icosahedral	150		2048	120	yes	128	85	78	no	[15]
Torroidal surface complex	Bacteriophage ϕ12	[31]	2011	744	8	2.6	0.88	1	C6	100		2048	300	yes	130	80	22	no	[8]
Chemotactic arrays	*E. coli*	[32]	2011	1098	5 to 8	3.3	0.75	1	C6	600	18	2048	300	yes	140	70-280	113	no	[15]
Bacterial flagellar motor, FliL-mutant	*Borrelia burgdorferi*	[33]	2011	711	5	4	0.56	1	1	300		4096	300	no	128	100	143	no	[2]
Envelope Glycoproteins	Trimeric Simian Immunodeficiency Virus	[34]	2010	4000	2.5	2	0.41	10	C3	100	34	2048	200	yes	130	90	41	no	[23]
GAG protein layer	HIV-1	[35]	2010		2.6 to 2.9	2.65	0.4	1	C6	150		2048	300	yes	120	90	38	no	[8]
GAG protein layer	Native HIV-1 budding sites	[36]	2010		6 to 8	4	0.713-0.82	1	C6			2048	300	yes	120	80	38	no	[8]
Photosystem II	Spinach chloroplasts	[37]	2011	100	8	4	0.58	1	1	150		2048	300	yes	136	80	32		[25] , [15]
GAG protein layer	HIV-1	[38]	2011		4 (first zero 3.7nm)	4	0.78			150		2048	120	yes	132	70	125		[12]
	RNA releasing poliovirus	[39]	2011	540	6	4.5	0.42	1	icosahedral	60	34	2048	300	yes	140	140	54	no	[5]
Double hairpin	Moloney murine leukemia virus	[40]	2010	38	8		0.65	2	1		23	4096	200	yes	120	90	10	no	[4]
Flagellar motor	*Treponema palladum*	[41]	2010	830	4 to 6	4	0.28	1	1	300		4096	300	no	130	100	143	no	[2]
Surface Layer	*Caulobacter crescentus*	[42]	2010	3777	3.6 to 12		0.69	2	C6	500		2048	300	yes	124	140	76	no	[8] and [43]
GAG protein layer	HIV	[44]	2010	9698	4.5	2.8	0.4	1	C6	150		2048	300	yes	120	70	38	no	[8]

Protein complex	Origin	Reference	Publication Year	N part	Under–focus [μm]	Reso–lution [nm]	Pixel size [nm]	Classes	Sym–metry	Ice thick–ness [nm]	mag [x1000]	Camer a [pixels]	Acceler ating voltage [keV]	Energy filter used?	Angular coverage [deg]	Total electron dose [e/A^2]	Largest linear size [nm]	Dualt ilt used ?	Data processing software
	HIV		2010	5438	2.1	2.55	0.4	1	C6	150		2048	300	yes	120	70	38	no	
	M-PVM		2010	10858	2.2	2.65	0.4	1	C6	150		2048	300	yes	120	70	38	no	
	RSV		2010	6732	2	2.3	0.4	1	C6	150		2048	300	yes	120	70	38	no	
Thermosomes		[11]	2010	3455	2.5		0.43	3	1	50		2048	160	no	120		34	no	[11]
Phage head head	Epsilon15 Bacteriophage	[45]	2010	50	0*	2.5		1	icosahed ral	150	30	2048	300	yes	140	80	100	no	[4]
	Epsilon15 Bacteriophage		2010	95	0*	3		1	1	150	30	2048	300	yes	140	80	100	no	
Multidrug efflux pump	*Pseudomonasaeruginosa*	*[46]*	2010	919	4	3		1	C3		27.5	2048	300	yes	138	60	21	no	[5]
Podovirus P-SSP7	*Prochlorococcus host cells*	*[47]*	2010	29				1	1			4096	300	no			50	no	
	Bacteriophage ϕ6	[48]	2010	315	4	3.7	0.78	1	1	50	38.5	2048	120	yes	140	75	50	no	[12]
	Bacteriophage BPP-1	[49]	2009	60			0.39					4096	300	no	140	100	68	no	
	Microtubule-kinesin complex	[50]	2010	99	6	3.2	0.76	1	helical 16	50	29	4096	200	no	140	100	130	no	[25]
Ribosomes	Human cells	[51]	2010	1911	6	3.9	0.82	2	1	200-400	17.5	2048	300	yes	130	85	30	no	[8]
ATP synthase	*Polytomella mitochondria*	*[52]*	2010	166	10	5.7	0.73	1	1			2048	300	yes		80	87	yes	[25]
Nuclear pore complex	*Xenopus*	*[53]*	2010	2488	4 to 6	6.4	0.65	1	1	200		2048	300	yes	124	-	150	no	[8]
Bacterial flagellar motor	*Treponema pallidum*	*[54]*	2009		15	10				300			400	yes	120	120		no	[5]
	Bovine papiloma virus	[55]	2009	519	9#	4		1	12	50			300	yes			50	no	[25]
Bacterial flagellar motor	*Borrelia burgdorferi*	[56]	2010	138	10	4.6	0.82	1	c16	300		2048	300	yes	120	200	100	no	[8]
	PRD1 bacteriophage	[57]	2009	311	3 to 5.6 (mean 4) #	2.2	0.47	1	icosahed ral	100		2048	300	yes	126	70	100	no	[8] and [12]
Bacterial flagellar motor	*Borrelia burgdorferi*	[58]	2009	1280	4 to 6	3.5	0.22	1	1	300		4096	300	no	130	100	100	no	[2]
	Borrelia burgdorferi, transposon mutant		2009	1100	4 to 6	3.5	0.22	1	1	300		4096	300	no	130	100	100	no	
	detergent treated *Borrelia Burgdoferii,* WT		2009	454	4 to 6	3	0.22	1	1	300		4096	300	no	130	100	50	no	
Triad Junctions		[59]	2009	49	10	7.1	0.5	3	C4	250		1024	200	no	120	-	30	no	[5]
Flagella	*Chlamydomonas,* WT	*[60]*	2008	559	2 to 4	4.1	0.7	1	1		19.3	2048	200	yes	120	-	96	no	[5] and [61]
	Chlamydomonas, oda1-		2008	656	2 to 4	3.8	0.7	1	1		19.3	2048	200	yes	120	-	96	no	
Chemotactic arrays, TsrQEQE	*E. coli*	[62]	2008	3972	5 to 8	3.3		2	c3	500	44	2048	300	yes	140	60-80	20	no	[15]
Chemotactic arrays TsrQEQE+serine	*E. coli*		2008	23505	5 to 8	3.3		1	c3	500	44	2048	300	yes	140	60-80	20	no	
Chemotactic arrays	*Caulobacter crescentus*	[63]	2008	1200	4 to 6		7.5	1	c6	350	18	2048	300	yes	140	45-75	47	no	

Protein complex	Origin	Reference	Publication Year	N part	Under–focus [µm]	Reso–lution [nm]	Pixel size [nm]	Classes	Sym–metry	Ice thick–ness [nm]	mag [x1000]	Camer a [pixels]	Acceler ating voltage [keV]	Energy filter used?	Angular coverage [deg]	Total electron dose [e/A^2]	Largest linear size [nm]	Dualt ilt used ?	Data processing software
gp120 trimers	HIV-1	[64]	2008		2	2.2	4.1	1	c3	150		2048	200	yes	140	80	15	no	[2]
ATP synthase dimers	rat liver	[65]	2008	235	10 to 15	5		1	1	160		2048	300	yes		100 - 200	40	no	[5]
	Simian Virus 40	[66]	2007	13	10	7	0.55	1	icosahed ral	100		2048	300	yes		55-75	50	no	[8] and [12]
Donut-shaped surface spike	cystovirus ϕ12	[67]	2008	1300	8#	4.6		1	1	150	43	4096	200	no	140	30-40	20	no	[5] and [2]
Cadherins in desmosomes		[68]	2007		4 to 9	3.4	0.6	1	1	50$		2048	300	yes	130	40	34	no	
Nuclear pore complex	*Dictyostelium discoideum*	*[69]*	2007	4184	12 to 15	5.8	0.82	1	1	500		2048	300	yes	126	NA	150	no	[8]
Outer dynein arms		[70]	2007	667	2 to 3	4.5		1	1	250		2048	200	yes	120	30	120	no	
ENV spike	SIV	[71]	2006	2986	4 to 6	2.8	0.55	1	c3	150		2048	300	yes	123	50 to 70	12	no	[8]
Portal	Herpes Simplex Virus	[72]	2006	150		5.5	0.78	1	c5	150	38	2048	120	yes	114	35 to 80	150	no	[12]
Nuclear pore complex	Isolated Xenopus nuclear envelope	[73]	2003	446	1.5	12		1	c8	200		1024	120	yes	120	20 to 28	150	no	[28]
	Hepes Simplex Virus	[74]	2003	11	8	5.6		1	icosahed ral	250			120	no	123	40	50	no	[12]
Envelope protein	Retrovirus	[8]	2005	1114	4 to 6	2.7	5.5	1	c3	100	55	2048	300	yes	132		20	no	[8]
Thermosome		[75]	1997	307		2	0.48	1	82 point group	50			120	no	101	20	20	no	[28]
GAG protein layer	Immature HIV	[76]	2009		2 to 5#	1.7	0.4	1	1	150		2048	300	yes	126	70	100	no	[8]
Matrix protein	Measles virus	[77]	2011	1400	3 to 6	4.4	0.38	1	1		39.4	2048	200	no	120		50	no	[78]
Radial spokes of cilia and flagella		[79]	2011	2400	3 to 5	3.9		1	1	220	19	2048	200	yes	120		96	no	[12] and [5]
Radial spokes in cilia and flagella		[80]	2012	1300	8	3.6	1	1	1	200	13.5	2048	300	yes		100	96	no	[25]
Bovine respirasome		[81]	2011	2466	2	2.2	0.38	1	1	50	78	2048	200	yes	130	40	30	no	[8] and [11]
Envelope Glycoproteins	Simian Immunodeficiency Virus	[22]	2011	4826	2.5	2.5	0.41	1	c3	100		2048	200	yes	125	70-140	41	no	[23]
Chromatin fibers		[82]	2011	1000	6.5 to 8	4.3	0.6	1	1	80$	22500	2048	300	yes	126	90	60	no	[83] and [84]
gp140	HIV-1	[85]	2011	4000	2.5	2	0.41	1	C3	100	34	2048	200	yes	130	90	41	no	[23]
Desmosomal plague		[86]	2011		4 to 9	3.2	0.6	1	1	50$		2048	300	yes	130	40	25	no	[8]
Envelope spikes	HIV-1	[87]	2011	1463	4 to 5	3.3	0.23	1	c3	170	39	4096	300	no	130	100	14	no	[2]
envelope spikes	AIDS virus	[88]	2006	6175	4 to 6	3.2	0.56	1	c3	150	43	2048	300	no	140	60	14	no	[2]
RNA Packaging Element	Retrovirus	[89]	2010	47		4	0.65	2	1	50	23	4096	200	yes	120		10	no	
	Sulfolobus turreted icosahedral virus	[90]	2010	123	10 to 14	6.5	1.26	2	icosahed ral	300	18	4096	300	yes	120	160	100	no	[91]
Hibernating ribosomes	Bacterial cell lyzates	[92]	2010	1232	3		0.28	1	1	100	54	4096	160	no	120	50	50	no	[8]

Protein complex	Origin	Reference	Publication Year	N part	Under–focus [µm]	Reso–lution [nm]	Pixel size [nm]	Classes	Sym–metry	Ice thick–ness [nm]	mag [x1000]	Camera [pixels]	Accelerating voltage [keV]	Energy filter used?	Angular coverage [deg]	Total electron dose [e/A^2]	Largest linear size [nm]	Dualtilt used?	Data processing software
	Intact starved E.coli		2010	601	9#	3	0.71	1	1	400	42	2048	300	yes	120		50	no	
Bacterial membrane Eflix pump		[46]	2010	919	4	3	0.82	1	1	100	27.5	2048	300	yes	138	60	30	no	[5]
PSII in chloroplast membranes		[93]	2010	300	6 to 10		0.71	1	2	150$		2048	300	yes	130	150	10	no	[25]
ATP synthase in chloroplast membranes			2010	50	6 to 10		0.71	1	1	150$		2048	300	yes	130	150	12	no	
	Bordetella bacteriophage	[49]	2010	60			0.4	1	icosahedral	100		4096	300	no	140	100	70	no	[2]
Dynein regulatory complex in cila and flagella		[94]	2009	720	6 to 8	3.3		1	1		13.5	2048	300	yes	130	100	96	no	[25]
Inner dynein arms and inter-doublet links in	*Chlamydomonas* flagella	[95]	2009	405	2 to 4							2048	200	yes	120		96	no	[12] and [5]
ATP synthase dimers		[52]	2009	550	10	5.7	0.7	1	c2	500	41.4	2048	300	yes		80	30	yes	[25]
Envelope Spikes	HIV-1	[96]	2008	2070			0.56	8	c3	100	43	2048	300	no	130	100	12	no	[2]
	Rift Valley Fever Virus	[97]	2008	46		6		1	icosahedral	150	15	4096	200		136		100	no	[15] and [4]
Integrin $\alpha_{IIb}\beta_3$ in Membrane Environment		[98]	2008	1715	6		0.56					2048	300	no	124		11	no	[2]
	Kaposi's sarcoma-associated herpesvirus	[99]	2008	297	8		0.89	1	1			4096	300		140		100	no	[100]
Receptor–membrane complex	Poliovirus	[101]	2007	1200	4	3	0.99	1	c5	150		2048	300	yes	132	100	50	no	[5] and [25]
26S proteasome		[102]	2006	153	2.5	3.5	0.39	1	c2	80	36		160	no	120	20	50	no	[61]
Capsid	Herpesvirus	[103]	2007	38		5.7		1	icosahedral	200	27.8		200	no	140	50	200	no	[4]
Glycoprotein spikes	Influenza	[104]	2006		4 to 6		0.78			150		2048	120	yes	136	55	20	no	
Axonemes	Chlamydomonas	[25]	2006	280	6 to 8	4	0.95	1	1	200		2048	300	yes	130	100	96	no	[25]
Bacterial flagellar motor	*Treponema primitia*	[105]	2006	20	10 to 18	7	0.98	1	c16	250		2048	300	yes	126	110	100	no	[12] and [18]
Myosin V (neg stain)		[106]	2006	4029	5 to 12#	2.4	0.56	1	C6	50	43	2048	300	no	140	30	40	yes	[5]
Virus core	Hepatitis B	[107]	2006	80	5.7#	2.4	5	1	icosahedral			2048	300	no	102			no	[12]
Pyruvate and 2-Oxoglutarate Dehydrogenase Complexes	*E. coli*	[108]	2005	305	10	5.5	0.82	1	octahedral	50	27.5	2048	300	yes	126	120	15	yes	[12] and [18]
Thermosome		[109]	1998	307	2	2.8	0.48	1	82 point group	50	39		120	no	101	20	20	no	[28]
Nuclear pore complex	*Dictyostelium discoideum*	[110]	2004	267	15	8.3	0.82		c8	500		2048	300	yes	126		150	no	[8]

Table S1. Protein complexes studied by cryo electron tomography and sub-tomogram averaging with acquisition parameters.

Remarks for Table S1:

- Each publication may contain several structures acquired at different imaging conditions, in such cases several rows are possible for one publication
- Publication year may mismatch with the values in the table due to advance publication date.
- In cases resolution was not explicitly specified but the Fourier Shell Correlation (FSC) curve was provided, we estimated the resolution from the intersection of the FSC curve with the 0.5 – line.
- For underfocus values, # indicates that CTF correction was applied, * indicates the use of a phase plate.
- Number of classes and symmetry was assumed to be 1 resp. p1, unless another value is specified.
- Accelerating voltage was assumed to be highest achievable for the specified microscope unless another value specified.
- Ice thickness is specified in some manuscripts, for others we estimated as minimal possible ice thickness the dimension needed for accommodating the samples.

References

1. Zhang K, Tong BA, Liu J, Li C (2012) A single-domain FlgJ contributes to flagellar hook and filament formation in the Lyme disease spirochete Borrelia burgdorferi. J Bacteriol 194: 866-874.

2. Winkler H (2007) 3D reconstruction and processing of volumetric data in cryo-electron tomography. J Struct Biol 157: 126-137.

3. Li S, Fernandez JJ, Marshall WF, Agard DA (2012) Three-dimensional structure of basal body triplet revealed by electron cryo-tomography. Embo J 31: 552-562.

4. Schmid MF, Booth CR (2008) Methods for aligning and for averaging 3D volumes with missing data. J Struct Biol 161: 243-248.

5. Frank J, Radermacher M, Penczek P, Zhu J, Li Y, et al. (1996) SPIDER and WEB: processing and visualization of images in 3D electron microscopy and related fields. J Struct Biol 116: 190-199.

6. Beniac DR, Melito PL, Devarennes SL, Hiebert SL, Rabb MJ, et al. (2012) The organisation of Ebola virus reveals a capacity for extensive, modular polyploidy. PLoS ONE 7: e29608.

7. Bharat TA, Riches JD, Kolesnikova L, Welsch S, Krahling V, et al. (2011) Cryo-electron tomography of Marburg virus particles and their morphogenesis within infected cells. PLoS Biol 9: e1001196.

8. Forster F, Medalia O, Zauberman N, Baumeister W, Fass D (2005) Retrovirus envelope protein complex structure in situ studied by cryo-electron tomography. Proc Natl Acad Sci U S A 102: 4729-4734.

9. Castano-Diez D, Kudryashev M, Arheit M, Stahlberg H (2012) Dynamo: A flexible, user-friendly development tool for subtomogram averaging of cryo-EM data in high-performance computing environments. J Struct Biol 178:139-151

10. Fontana J, Cardone G, Heymann JB, Winkler DC, Steven AC (2012) Structural Changes in Influenza Virus at Low pH Characterized by Cryo-Electron Tomography. Journal of virology 86: 2919-2929.

11. Stolken M, Beck F, Haller T, Hegerl R, Gutsche I, et al. (2011) Maximum likelihood based classification of electron tomographic data. J Struct Biol 173: 77-85.

12. Heymann JB, Cardone G, Winkler DC, Steven AC (2008) Computational resources for cryo-electron tomography in Bsoft. J Struct Biol 161: 232-242.

13. Ueno H, Ishikawa T, Bui KH, Gonda K, Yamaguchi T (2012) Mouse respiratory cilia with the asymmetric axonemal structure on sparsely distributed ciliary cells can generate overall directional flow. Nanomedicine. doi:10.1016/j.nano.2012.01.004

14. Koyfman AY, Schmid MF, Gheiratmand L, Fu CJ, Khant HA, et al. (2011) Structure of Trypanosoma brucei flagellum accounts for its bihelical motion. Proc Natl Acad Sci U S A 108: 11105-11108.

15. Ludtke SJ, Baldwin PR, Chiu W (1999) EMAN: semiautomated software for high-resolution single-particle reconstructions. J Struct Biol 128: 82-97.

16. Wu S, Liu J, Reedy MC, Tregear RT, Winkler H, et al. (2010) Electron tomography of cryofixed, isometrically contracting insect flight muscle reveals novel actin-myosin interactions. PLoS ONE 5: e12643. doi:10.1371/journal.pone.0012643

17. Chen S, Beeby M, Murphy GE, Leadbetter JR, Hendrixson DR, et al. (2011) Structural diversity of bacterial flagellar motors. Embo J 30: 2972-2981.

18. Leong PA, Heymann JB, Jensen GJ (2005) Peach: a simple Perl-based system for distributed computation and its application to cryo-EM data processing. Structure 13: 505-511.

19. Barber CF, Heuser T, Carbajal-Gonzalez BI, Botchkarev VV, Jr., Nicastro D (2012) Three-dimensional structure of the radial spokes reveals heterogeneity and interactions with dyneins in Chlamydomonas flagella. Mol Biol Cell 23: 111-120.

20. Nemecek D, Cheng N, Qiao J, Mindich L, Steven AC, et al. (2011) Stepwise expansion of the bacteriophage varphi6 procapsid: possible packaging intermediates. Journal of molecular biology 414: 260-271.

21. Fu X, Walter MH, Paredes A, Morais MC, Liu J (2011) The mechanism of DNA ejection in the Bacillus anthracis spore-binding phage 8a revealed by cryo-electron tomography. Virology 421: 141-148.

22. White TA, Bartesaghi A, Borgnia MJ, de la Cruz MJ, Nandwani R, et al. (2011) Three-dimensional structures of soluble CD4-bound states of trimeric simian immunodeficiency virus envelope glycoproteins determined by using cryo-electron tomography. Journal of virology 85: 12114-12123.

23. Bartesaghi A, Sprechmann P, Liu J, Randall G, Sapiro G, et al. (2008) Classification and 3D averaging with missing wedge correction in biological electron tomography. J Struct Biol 162: 436-450.

24. Nicastro D, Fu X, Heuser T, Tso A, Porter ME, et al. (2011) Cryo-electron tomography reveals conserved features of doublet microtubules in flagella. Proc Natl Acad Sci U S A 108: E845-853.

25. Nicastro D, Schwartz C, Pierson J, Gaudette R, Porter ME, et al. (2006) The molecular architecture of axonemes revealed by cryoelectron tomography. Science 313: 944-948.

26. Sui H, Downing KH (2006) Molecular architecture of axonemal microtubule doublets revealed by cryo-electron tomography. Nature 442: 475-478.

27. Nicastro D, McIntosh JR, Baumeister W (2005) 3D structure of eukaryotic flagella in a quiescent state revealed by cryo-electron tomography. Proc Natl Acad Sci U S A 102: 15889-15894.

28. Hegerl R (1996) The EM Program Package: A Platform for Image Processing in Biological Electron Microscopy. J Struct Biol 116: 30-34.

29. Liu J, Chen CY, Shiomi D, Niki H, Margolin W (2011) Visualization of bacteriophage P1 infection by cryo-electron tomography of tiny Escherichia coli. Virology 417: 304-311.

30. Plevka P, Battisti AJ, Junjhon J, Winkler DC, Holdaway HA, et al. (2011) Maturation of flaviviruses starts from one or more icosahedrally independent nucleation centres. Embo rep 12: 602-606.

31. Leo-Macias A, Katz G, Wei H, Alimova A, Katz A, et al. (2011) Toroidal surface complexes of bacteriophage varphi12 are responsible for host-cell attachment. Virology 414: 103-109.

32. Khursigara CM, Lan G, Neumann S, Wu X, Ravindran S, et al. (2011) Lateral density of receptor arrays in the membrane plane influences sensitivity of the E. coli chemotaxis response. Embo J 30: 1719-1729.

33. Motaleb MA, Pitzer JE, Sultan SZ, Liu J (2011) A novel gene inactivation system reveals altered periplasmic flagellar orientation in a Borrelia burgdorferi fliL mutant. J Bacteriol 193: 3324-3331.

34. White TA, Bartesaghi A, Borgnia MJ, Meyerson JR, de la Cruz MJ, et al. (2010) Molecular architectures of trimeric SIV and HIV-1 envelope glycoproteins on intact viruses: strain-dependent variation in quaternary structure. PLoS pathogens 6: e1001249.

35. de Marco A, Muller B, Glass B, Riches JD, Krausslich HG, et al. (2010) Structural analysis of HIV-1 maturation using cryo-electron tomography. PLoS pathogens 6: e1001215.

36. Carlson LA, de Marco A, Oberwinkler H, Habermann A, Briggs JA, et al. (2010) Cryo electron tomography of native HIV-1 budding sites. PLoS pathogens 6: e1001173.

37. Kouril R, Oostergetel GT, Boekema EJ (2011) Fine structure of granal thylakoid membrane organization using cryo electron tomography. Biochimica et biophysica acta 1807: 368-374.

38. Keller PW, Adamson CS, Heymann JB, Freed EO, Steven AC (2011) HIV-1 maturation inhibitor bevirimat stabilizes the immature Gag lattice. Journal of virology 85: 1420-1428.

39. Bostina M, Levy H, Filman DJ, Hogle JM (2011) Poliovirus RNA is released from the capsid near a twofold symmetry axis. Journal of virology 85: 776-783.

40. Miyazaki Y, Garcia EL, King SR, Iyalla K, Loeliger K, et al. (2010) An RNA structural switch regulates diploid genome packaging by Moloney murine leukemia virus. Journal of molecular biology 396: 141-152.

41. Liu J, Howell JK, Bradley SD, Zheng Y, Zhou ZH, et al. (2010) Cellular architecture of Treponema pallidum: novel flagellum, periplasmic cone, and cell envelope as revealed by cryo electron tomography. Journal of molecular biology 403: 546-561.

42. Amat F, Comolli LR, Nomellini JF, Moussavi F, Downing KH, et al. (2010) Analysis of the intact surface layer of Caulobacter crescentus by cryo-electron tomography. J Bacteriol 192: 5855-5865.

43. Amat F, Comolli LR, Moussavi F, Smit J, Downing KH, et al. (2010) Subtomogram alignment by adaptive Fourier coefficient thresholding. Journal of structural biology 171: 332-344.

44. de Marco A, Davey NE, Ulbrich P, Phillips JM, Lux V, et al. (2010) Conserved and variable features of Gag structure and arrangement in immature retrovirus particles. Journal of virology 84: 11729-11736.

45. Murata K, Liu X, Danev R, Jakana J, Schmid MF, et al. (2010) Zernike phase contrast cryo-electron microscopy and tomography for structure determination at nanometer and subnanometer resolutions. Structure 18: 903-912.

46. Trepout S, Taveau JC, Benabdelhak H, Granier T, Ducruix A, et al. (2010) Structure of reconstituted bacterial membrane efflux pump by cryo-electron tomography. Biochimica et biophysica acta 1798: 1953-1960.

47. Liu X, Zhang Q, Murata K, Baker ML, Sullivan MB, et al. (2010) Structural changes in a marine podovirus associated with release of its genome into Prochlorococcus. Nature structural & molecular biology 17: 830-836.

48. Nemecek D, Heymann JB, Qiao J, Mindich L, Steven AC (2010) Cryo-electron tomography of bacteriophage phi6 procapsids shows random occupancy of the binding sites for RNA polymerase and packaging NTPase. J Struct Biol 171: 389-396.

49. Dai W, Hodes A, Hui WH, Gingery M, Miller JF, et al. (2010) Three-dimensional structure of tropism-switching Bordetella bacteriophage. Proc Natl Acad Sci U S A 107: 4347-4352.

50. Cope J, Gilbert S, Rayment I, Mastronarde D, Hoenger A (2010) Cryo-electron tomography of microtubule-kinesin motor complexes. J Struct Biol 170: 257-265.

51. Brandt F, Carlson LA, Hartl FU, Baumeister W, Grunewald K (2010) The three-dimensional organization of polyribosomes in intact human cells. Molecular cell 39: 560-569.

52. Dudkina NV, Oostergetel GT, Lewejohann D, Braun HP, Boekema EJ (2010) Row-like organization of ATP synthase in intact mitochondria determined by cryo-electron tomography. Biochimica et biophysica acta 1797: 272-277.

53. Frenkiel-Krispin D, Maco B, Aebi U, Medalia O (2010) Structural analysis of a metazoan nuclear pore complex reveals a fused concentric ring architecture. Journal of molecular biology 395: 578-586.

54. Izard J, Renken C, Hsieh CE, Desrosiers DC, Dunham-Ems S, et al. (2009) Cryo-electron tomography elucidates the molecular architecture of Treponema pallidum, the syphilis spirochete. J Bacteriol 191: 7566-7580.

55. Xiong Q, Morphew MK, Schwartz CL, Hoenger AH, Mastronarde DN (2009) CTF determination and correction for low dose tomographic tilt series. Journal of structural biology 168: 378-387.

56. Kudryashev M, Cyrklaff M, Wallich R, Baumeister W, Frischknecht F (2010) Distinct in situ structures of the Borrelia flagellar motor. J Struct Biol 169: 54-61.

57. Zanetti G, Riches JD, Fuller SD, Briggs JA (2009) Contrast transfer function correction applied to cryo-electron tomography and sub-tomogram averaging. Journal of structural biology 168: 305-312.

58. Liu J, Lin T, Botkin DJ, McCrum E, Winkler H, et al. (2009) Intact flagellar motor of Borrelia burgdorferi revealed by cryo-electron tomography: evidence for stator ring curvature and rotor/C-ring assembly flexion. J Bacteriol 191: 5026-5036.

59. Renken C, Hsieh CE, Marko M, Rath B, Leith A, et al. (2009) Structure of frozen-hydrated triad junctions: a case study in motif searching inside tomograms. J Struct Biol 165: 53-63.

60. Bui KH, Sakakibara H, Movassagh T, Oiwa K, Ishikawa T (2008) Molecular architecture of inner dynein arms in situ in Chlamydomonas reinhardtii flagella. J Cell Biol 183: 923-932.

61. Nickell S, Forster F, Linaroudis A, Net WD, Beck F, et al. (2005) TOM software toolbox: acquisition and analysis for electron tomography. J Struct Biol 149: 227-234.

62. Khursigara CM, Wu X, Zhang P, Lefman J, Subramaniam S (2008) Role of HAMP domains in chemotaxis signaling by bacterial chemoreceptors. Proc Natl Acad Sci U S A 105: 16555-16560.

63. Khursigara CM, Wu X, Subramaniam S (2008) Chemoreceptors in Caulobacter crescentus: trimers of receptor dimers in a partially ordered hexagonally packed array. J Bacteriol 190: 6805-6810.

64. Liu J, Bartesaghi A, Borgnia MJ, Sapiro G, Subramaniam S (2008) Molecular architecture of native HIV-1 gp120 trimers. Nature 455: 109-113.

65. Strauss M, Hofhaus G, Schroder RR, Kuhlbrandt W (2008) Dimer ribbons of ATP synthase shape the inner mitochondrial membrane. Embo J 27: 1154-1160.

66. Schelhaas M, Malmstrom J, Pelkmans L, Haugstetter J, Ellgaard L, et al. (2007) Simian Virus 40 depends on ER protein folding and quality control factors for entry into host cells. Cell 131: 516-529.

67. Hu GB, Wei H, Rice WJ, Stokes DL, Gottlieb P (2008) Electron cryo-tomographic structure of cystovirus phi 12. Virology 372: 1-9.

68. Al-Amoudi A, Diez DC, Betts MJ, Frangakis AS (2007) The molecular architecture of cadherins in native epidermal desmosomes. Nature 450: 832-837.

69. Beck M, Lucic V, Forster F, Baumeister W, Medalia O (2007) Snapshots of nuclear pore complexes in action captured by cryo-electron tomography. Nature 449: 611-615.

70. Ishikawa T, Sakakibara H, Oiwa K (2007) The architecture of outer dynein arms in situ. Journal of molecular biology 368: 1249-1258.

71. Zanetti G, Briggs JA, Grunewald K, Sattentau QJ, Fuller SD (2006) Cryo-electron tomographic structure of an immunodeficiency virus envelope complex in situ. PLoS pathogens 2: e83.

72. Cardone G, Winkler DC, Trus BL, Cheng N, Heuser JE, et al. (2007) Visualization of the herpes simplex virus portal in situ by cryo-electron tomography. Virology 361: 426-434.

73. Stoffler D, Feja B, Fahrenkrog B, Walz J, Typke D, et al. (2003) Cryo-electron tomography provides novel insights into nuclear pore architecture: implications for nucleocytoplasmic transport. Journal of molecular biology 328: 119-130.

74. Grunewald K, Desai P, Winkler DC, Heymann JB, Belnap DM, et al. (2003) Three-dimensional structure of herpes simplex virus from cryo-electron tomography. Science 302: 1396-1398.

75. Walz J, Typke D, Nitsch M, Koster AJ, Hegerl R, et al. (1997) Electron Tomography of Single Ice-Embedded Macromolecules: Three-Dimensional Alignment and Classification. J Struct Biol 120: 387-395.

76. Briggs JA, Riches JD, Glass B, Bartonova V, Zanetti G, et al. (2009) Structure and assembly of immature HIV. Proc Natl Acad Sci U S A 106: 11090-11095.

77. Liljeroos L, Huiskonen JT, Ora A, Susi P, Butcher SJ (2011) Electron cryotomography of measles virus reveals how matrix protein coats the ribonucleocapsid within intact virions. Proc Natl Acad Sci U S A 108: 18085-18090.

78. Huiskonen JT, Hepojoki J, Laurinmaki P, Vaheri A, Lankinen H, et al. (2010) Electron cryotomography of Tula hantavirus suggests a unique assembly paradigm for enveloped viruses. Journal of virology 84: 4889-4897.

79. Pigino G, Bui KH, Maheshwari A, Lupetti P, Diener D, et al. (2011) Cryoelectron tomography of radial spokes in cilia and flagella. J Cell Biol 195: 673-687.

80. Lin J, Heuser T, Carbajal-Gonzalez BI, Song K, Nicastro D (2012) The structural heterogeneity of radial spokes in cilia and flagella is conserved. Cytoskeleton (Hoboken) 69: 88-100.

81. Dudkina NV, Kudryashev M, Stahlberg H, Boekema EJ (2011) Interaction of complexes I, III, and IV within the bovine respirasome by single particle cryoelectron tomography. Proceedings of the National Academy of Sciences of the United States of America 108: 15196-15200.

82. Scheffer MP, Eltsov M, Frangakis AS (2011) Evidence for short-range helical order in the 30-nm chromatin fibers of erythrocyte nuclei. Proc Natl Acad Sci U S A 108: 16992-16997.

83. Forster F, Pruggnaller S, Seybert A, Frangakis AS (2008) Classification of cryo-electron sub-tomograms using constrained correlation. J Struct Biol 161: 276-286.

84. Yu Z, Frangakis AS (2011) Classification of electron sub-tomograms with neural networks and its application to template-matching. J Struct Biol 174: 494-504.

85. Harris A, Borgnia MJ, Shi D, Bartesaghi A, He H, et al. (2011) Trimeric HIV-1 glycoprotein gp140 immunogens and native HIV-1 envelope glycoproteins display the same closed and open quaternary molecular architectures. Proc Natl Acad Sci U S A 108: 11440-11445.

86. Al-Amoudi A, Castano-Diez D, Devos DP, Russell RB, Johnson GT, et al. (2011) The three-dimensional molecular structure of the desmosomal plaque. Proceedings of the National Academy of Sciences of the United States of America 108: 6480-6485.

87. Hu G, Liu J, Taylor KA, Roux KH (2011) Structural comparison of HIV-1 envelope spikes with and without the V1/V2 loop. Journal of virology 85: 2741-2750.

88. Zhu P, Liu J, Bess J, Jr., Chertova E, Lifson JD, et al. (2006) Distribution and three-dimensional structure of AIDS virus envelope spikes. Nature 441: 847-852.

89. Miyazaki Y, Irobalieva RN, Tolbert BS, Smalls-Mantey A, Iyalla K, et al. (2010) Structure of a conserved retroviral RNA packaging element by NMR spectroscopy and cryo-electron tomography. Journal of molecular biology 404: 751-772.

90. Fu CY, Wang K, Gan L, Lanman J, Khayat R, et al. (2010) In vivo assembly of an archaeal virus studied with whole-cell electron cryotomography. Structure 18: 1579-1586.

91. Yin Z, Zheng Y, Doerschuk PC, Natarajan P, Johnson JE (2003) A statistical approach to computer processing of cryo-electron microscope images: virion classification and 3-D reconstruction. J Struct Biol 144: 24-50.

92. Ortiz JO, Brandt F, Matias VR, Sennels L, Rappsilber J, et al. (2010) Structure of hibernating ribosomes studied by cryoelectron tomography in vitro and in situ. The Journal of cell biology 190: 613-621.

93. Daum B, Nicastro D, Austin J, 2nd, McIntosh JR, Kuhlbrandt W (2010) Arrangement of photosystem II and ATP synthase in chloroplast membranes of spinach and pea. Plant Cell 22: 1299-1312.

94. Heuser T, Raytchev M, Krell J, Porter ME, Nicastro D (2009) The dynein regulatory complex is the nexin link and a major regulatory node in cilia and flagella. J Cell Biol 187: 921-933.

95. Bui KH, Sakakibara H, Movassagh T, Oiwa K, Ishikawa T (2009) Asymmetry of inner dynein arms and inter-doublet links in Chlamydomonas flagella. J Cell Biol 186: 437-446.

96. Zhu P, Winkler H, Chertova E, Taylor KA, Roux KH (2008) Cryoelectron tomography of HIV-1 envelope spikes: further evidence for tripod-like legs. PLoS pathogens 4: e1000203.

97. Freiberg AN, Sherman MB, Morais MC, Holbrook MR, Watowich SJ (2008) Three-dimensional organization of Rift Valley fever virus revealed by cryoelectron tomography. Journal of virology 82: 10341-10348.

98. Ye F, Liu J, Winkler H, Taylor KA (2008) Integrin alpha IIb beta 3 in a membrane environment remains the same height after Mn2+ activation when observed by cryoelectron tomography. Journal of molecular biology 378: 976-986.

99. Deng B, O'Connor CM, Kedes DH, Zhou ZH (2008) Cryo-electron tomography of Kaposi's sarcoma-associated herpesvirus capsids reveals dynamic scaffolding structures essential to capsid assembly and maturation. J Struct Biol 161: 419-427.

100. Jiang W, Baker ML, Ludtke SJ, Chiu W (2001) Bridging the information gap: computational tools for intermediate resolution structure interpretation. Journal of molecular biology 308: 1033-1044.

101. Bostina M, Bubeck D, Schwartz C, Nicastro D, Filman DJ, et al. (2007) Single particle cryoelectron tomography characterization of the structure and structural variability of poliovirus-receptor-membrane complex at 30 A resolution. J Struct Biol 160: 200-210.

102. Nickell S, Beck F, Korinek A, Mihalache O, Baumeister W, et al. (2007) Automated cryoelectron microscopy of "single particles" applied to the 26S proteasome. FEBS Lett 581: 2751-2756.

103. Chang JT, Schmid MF, Rixon FJ, Chiu W (2007) Electron cryotomography reveals the portal in the herpesvirus capsid. Journal of virology 81: 2065-2068.

104. Harris A, Cardone G, Winkler DC, Heymann JB, Brecher M, et al. (2006) Influenza virus pleiomorphy characterized by cryoelectron tomography. Proc Natl Acad Sci U S A 103: 19123-19127.

105. Murphy GE, Leadbetter JR, Jensen GJ (2006) In situ structure of the complete Treponema primitia flagellar motor. Nature 442: 1062-1064.

106. Liu J, Taylor DW, Krementsova EB, Trybus KM, Taylor KA (2006) Three-dimensional structure of the myosin V inhibited state by cryoelectron tomography. Nature 442: 208-211.

107. Fernandez JJ, Li S, Crowther RA (2006) CTF determination and correction in electron cryotomography. Ultramicroscopy 106: 587-596.

108. Murphy GE, Jensen GJ (2005) Electron cryotomography of the E. coli pyruvate and 2-oxoglutarate dehydrogenase complexes. Structure 13: 1765-1773.

109. Nitsch M, Walz J, Typke D, Klumpp M, Essen LO, et al. (1998) Group II chaperonin in an open conformation examined by electron tomography. Nat Struct Biol 5: 855-857.

110. Beck M, Forster F, Ecke M, Plitzko JM, Melchior F, et al. (2004) Nuclear pore complex structure and dynamics revealed by cryoelectron tomography. Science 306: 1387-1390.
